# Selenium Nanoparticles Decorated With Stevioside Potentially Attenuate Fructose Palmitate Induced Lipid Accumulation in HepG2 Cells

**DOI:** 10.1155/mi/7942947

**Published:** 2025-02-13

**Authors:** Shuai Li, Hui Yang, Wenjun Zhou, Ruoting Wang, Likang Li, Changfa Zhang, Jingyi Zhang, Yingxin Liu, Zhi Huang, Guowei Li

**Affiliations:** ^1^Medical Department, Jingchu University of Technology, Jingmen, China; ^2^Center for Clinical Epidemiology and Methodology (CCEM), The Affiliated Guangdong Second Provincial General Hospital of Jinan University, Guangzhou, China; ^3^Department of Health Management of the Guangdong Second Provincial General Hospital and Postdoctoral Research Station of Basic Medicine of the School of Medicine, Jinan University, Guangzhou, China; ^4^Department of Biotechnology, College of Life Science and Technology, Jinan University, Guangzhou, China; ^5^Department of Health Research Methods, Evidence, and Impact (HEI), McMaster University, 1280 Main St West, Hamilton, Ontario, Canada

**Keywords:** HepG2 cells, lipid accumulation, MAFLD, oxidative stress, selenium nanoparticles, stevioside

## Abstract

The excessive accumulation of lipid droplets within hepatocytes stands as a hallmark characteristic of metabolic-associated fatty liver disease (MAFLD). Selenium (Se) nanoparticles (NPs) have garnered considerable attention for their notable bioavailability, minimal toxicity, and exceptional antioxidant properties. However, a critical limitation lies in the propensity of SeNPs to aggregate into the biologically inactive elemental Se, thereby constraining their utility. Here, we utilized *Stevioside* (*SV*), a natural sweetener, to modify SeNPs and obtained the SV-SeNPs with a size of about 187 ± 7 nm. We aimed to investigate the effect of SV-SeNPs on high fructose–palmitate (HFP) induced lipid accumulation in HepG2 cells. Noteworthy is the absence of overt cytotoxicity attributed to SV-SeNPs on normal HepG2 cells. Of significance, our findings delineate the profound inhibitory effects of SV-SeNPs on the expression of key genes implicated in de novo lipogenesis, such as fatty-acid synthase (FASN), acetyl-CoA-carboxylase 1 (ACC1), and stearoyl-CoA desaturase–1 (SCD1) within HFP-induced HepG2 cells. Furthermore, our investigation reveals that SV-SeNPs mediate a significant reduction in lipid accumulation by activating the PI3K/AKT/Nrf2 signaling cascades. Additionally, the antioxidative properties of SV-SeNPs are underscored by their ability to counteract oxidative stress via the upregulation of two pivotal antioxidant enzymes, superoxide dismutase (SOD) and glutathione peroxidase (GSHPx). In conclusion, our study unveils the potential beneficial effects of SV-SeNPs on the prevention and treatment of MAFLD by effectively suppressing lipid accumulation and ameliorating oxidative stress.

## 1. Introduction

Metabolic-associated fatty liver disease (MAFLD), formerly known as nonalcoholic fatty liver disease (NAFLD) [1], is characterized by excessive fat accumulation (>5%) in the liver without a clear cause, such as alcohol use, virus infection, or drug-induced liver disease [2]. MAFLD is the most common liver disease worldwide, accounting for ~25% of the global population [3]. Obesity, type 2 diabetes mellitus and lipid metabolism dysfunction are significant risk factors for MAFLD [4]. The etiology and pathology of MAFLD are complex, heterogeneous, and still not completely understood. The prevailing theory is the “two-hit” hypothesis, with the initial accumulation of triglyceride (TG) and free fatty acids as the “first hit,” followed by inflammation, oxidative stress, and apoptosis as the “second hit” [5–7].

Selenium (Se) is an essential trace element with antioxidant properties, carrying out pleiotropic functions primarily through the 21st amino acid [8]. It is recognized as a natural ally against liver disease and a protective factor against liver necrosis. Research indicates that Se levels are lower in patients with MAFLD compared to healthy individuals [9], and decreased Se levels can lead to the buildup of lipid peroxides [10]. Se nanoparticles (NPs) have the advantages of high biocompatibility, high bioavailability, and low toxicity compared with the conventional supplement forms of selenate and are more effective than other forms of Se in increasing selenoprotein expression, scavenging free radicals [8, 11]. However, SeNPs are also unstable in liquid from, leading to aggregate into gray or black Se, thereby diminishing their biological activity and bioavailability [12]. Previous studies have shown that various biological macromolecules like proteins, polysaccharides, and polyphenols can modify SeNPs [13]. The modified ones cannot only manage the size and stability of SeNPs but also strengthen cellular uptake and prolong the circulation of SeNPs [14].

Reducing the consumption of sugary beverages and total fructose intake has a significant benefit in decreasing hepatic fat accumulation [15]. Noncaloric sweeteners can mimic the sweetness of sugar and help mitigate the risks of nutritional metabolic diseases [16]. Stevioside (SV), the primary sweetener in *Stevia rebaudiana* leaves, a plant species in the genus Stevia of the family Asteraceae, is a diterpene glycoside linked to glucose at C-4 and a disaccharide at C-13. The sweetening power of SV is estimated to be about 300-times than sucrose and is associated with beneficial biological effects such as anti-inflammatory, antioxidant, and lipid-lowering properties [17]. However, studies in both metabolic and animal have found that SV is poorly absorbed in the upper gastrointestinal tract and bloodstream [18], thus impacting its biological functions. SeNPs, with their high load capacity and ability to traverse biological barriers, may aid in drug delivery. In our preliminary experiments, it was observed that SV can modify SeNPs, and these SV-modified SeNPs remain stable with good biological activities.

Based on our hypothesis, SV-functionalized SeNPs could potentially offer antilipid aggregation and antioxidant effects, aiding in the prevention and treatment of MAFLD. To test this theory, we utilized SV as a surface modifier to enhance SeNPs, creating stable SV-SeNPs with an average diameter <200 nm. Subsequently, we examined its effect on lipid deposition induced by high fructose–palmitate (HFP) in HepG2 cells. Finally, we evaluated the influence of SV-SeNP on Nrf2/PI3K/AKT signaling pathway and oxidative stress in this cell model to provide insights for the prevention and treatment of MAFLD.

## 2. Materials and Methods

### 2.1. Chemicals and Reagents

SV (HY-N0669), PEG300 (HY-Y0873), and Tween-80 (HY-Y1891) were purchased from MedChemExpress. L-ascorbic acid (Vc) was provided by Sigma–Aldrich (St. Louis, USA). The sodium selenite (Na_2_SeO_3_) of analytical grade was supplied by Guangdong Guanghua Sci-Tech (Huada and JHD, Guangzhou, China), and the ultrapure Milli-Q water from Millipore (Bedford, MA, USA) was used in all experiments.

### 2.2. Synthesis of SV-SeNPs

First, a 10 mM SV solution was prepared by sequentially adding 10% DMSO, 40% PEG300, 5% Tween 80%, and 45% saline to the SV powder. The SV solution underwent ultrasonic treatment at 40 kHz for 30 min in an ultrasonic water bath to facilitate complete dissolution. Subsequently, 0.25 mL of 0.5% chitosan (CS) was mixed with 8 mL SV solution (10 mM) on an agitator at room temperature for 8 h to create a colloidal shell. Prior to adding 0.2 mL Na_2_SeO_3_ (100 mM), the mixture underwent dialysis in Milli-Q water for 12 h. With under magnetic stirring conditions, 1.5 mL Vc (100 mM) was added dropwise to the solution for the reduction reaction and stirred at room temperature for 12 more hours. Finally, the solution was dialyzed against Milli-Q water 24 h to remove any excess chemicals.

### 2.3. Characterization of SV-SeNPs

The size and morphology of SV-SeNPs were characterized using transmission electron microscope (TEM, Tecnai-10 Philips system), coupled with energy dispersive X-ray spectroscopy (EDS) for elemental analysis. Size distribution and zeta potential of MRO-SeNPs were determined utilizing a Zetasizer particle analyzer (Malvern Instruments Ltd.). Fourier-transform infrared (FT-IR) spectroscopy (Thermo Fisher Scientific, USA) spectra of SV-SeNPs were analyzed covering the range of 4000–400 cm^−1^. The stability of SV-SeNPs in various media, including phosphate buffered saline (PBS), fetal bovine serum (FBS), Dulbecco's modified eagle medium (DMEM), and water, was evaluated through dynamic light scattering (DLS, Nano ZS, Malvern Instruments, UK).

### 2.4. Cell Culture

The HepG2 cell line was purchased from ATCC (Shenzhen, China) and was cultured in DMEM supplemented with 10% FBS (Gibco, Gaithersburg, MD), 1% penicillin-streptomycin. Cells were maintained in a humidified incubator with 5% CO_2_ at 37°C. HepG2 cells were seeded at a density of 1 × 10^5^ cells per well in appropriate culture vessels. Cells were then divided into control and experimental groups based on the treatment conditions.

### 2.5. Cell Viability Assay

The cell viability was measured using a Cell Counting kit-8 (CCK-8) (K1018, APExBIO). The HepG2 cells were seeded in 96-well plates at a density of 2 × 10^3^ cells/well and incubated overnight in DMEM containing 10% FBS. Cells were incubated with BSA, HFP (100 mM fructose and 100 μM palmitate), HFP + SeNPs (2.5 µМ), HFP + SV-SeNPs (2.5 µМ), HFP + SeNPs (5 µМ), HFP + SV-SeNPs (5 µМ) for 48 h and were then incubated with 10 µL CCK-8 reagent for 2 h at 37°C. Absorbance at 450 nm was detected using an enzyme-labeled instrument (Multiskan Sky, Thermo Scientific) [19].

### 2.6. Oil Red O Staining

Oil Red O was used to detect the accumulation of lipid droplets in HepG2 cells. The HepG2 cells were seeded in six-well plates at a density of 1 × 10^5^ cells per well for 24 h. After respective treatments, the cells were washed twice with PBS and fixed them with ORO Fixativ for 30 min. Isopropanol (60%) was added, and the cells were soaked for 20 s. Following the removal of isopropanol, the cells were exposed to the oil red O dyeing solution and immersed for 15 min. Subsequently, Mayer's hematoxylin staining solution was added to counterstain the nuclei for 1 min, and then the lipid droplets were observed under a microscope [20].

### 2.7. Measurement of the TG Content

HFP-induced-HepG2 cells were treated with different concentrations of SeNPs and SV-SeNPs for 48 h. The HepG2 cell samples were collected and homogenized by ultrasonication for 5 min on ice. Centrifuge samples at 3000 rpm for 5 min and collect the supernatant. TG content was measured using a commercial kit (A110-1-1, Nanjing Jiancheng Bioengineering Institute) according to the manufacturer's protocol.

### 2.8. Superoxide Dismutase (SOD) Activity Measurement

HFP-induced-HepG2 cells were treated with different concentrations of SeNPs and SV-SeNPs for 48 h. Aspirate the cell culture medium, wash once with prechilled PBS at 4°C, and add the provided SOD sample preparation solution from the kit at a ratio of 100–200 µL per 1 × 10^6^ cells. Gently pipette to fully lyse the cells, then centrifuge at 4°C, 12,000 g for 5 min, and collect the supernatant as the sample to be tested. Subsequently, we used the commercial WST-8 method to determine the SOD level and operated according to instructions (S0101S, Beyotime).

### 2.9. RNA Isolation, cDNA Synthesis, and Real-Time PCR

Total RNA was isolated using a TaKaRa MiniBEST Universal RNA Extraction Kit (9767, Takara) according to the manufacturer's instructions. Total RNA was reverse transcribed and the relative mRNA levels of acetyl-CoA carboxylase 1 (ACC1), sterol regulatory element-binding protein 1 (SREBP1), stearoyl-CoA desaturase 1 (SCD1), fatty-acid synthase (FASN), peroxisome proliferator-activated receptor *α* (PPAR*α*), and PPAR*γ* were determined using a real-time quantitative PCR kit (TB Green Premix Ex Taq, RR420A, Takara) on a 7500 Real-Time PCR System (ThermoFisher). All the primers (Table S2) were synthesized from Sangon Biotech (Shanghai) Co., Ltd.

### 2.10. Western Blot Analysis

Cells were collected and lysed with RIPA buffer (P0013, Beyotime) containing protease inhibitors PMSF (ST506, Beyotime). The total protein concentration was established using a BCA quantification kit (P0010S, Beyotime). Samples containing equal amounts of protein were resolved by SDS-PAGE and then transferred to PVDF membranes (ISEQ00010, Merck Millipore). The membranes were blocked with 5% skim milk solution (P0216, Beyotime) at room temperature for 1 h and then incubated with primary antibodies (Table S1) overnight at 4°C. The membrane was rinsed three times for 5 min with TBST buffer followed by incubation with HRP-conjugated secondary antibody (Table S1) for 2 h at room temperature. The protein bands of interest were visualized using a BeyoECL Plus kit (P0018S, Beyotime) and automatic chemiluminescence image analysis system (Alliance Q9, UVITEC). The staining intensity of the bands was determined and analyzed using Quantity One software (Bio-Rad).

### 2.11. Statistical Analysis

Statistical analysis was performed using GraphPad Prism 7 software. Continuous data were presented as mean ± SEM. Comparisons among groups were performed using appropriate statistical tests depending on the study design and data distribution. For comparisons between two groups, unpaired *T*-tests or independent samples *T*-tests were utilized based on the normality and variance assumptions. For analyses involving more than two groups, one-way analysis of variance (ANOVA) was employed. Post-hoc tests, such as Tukey's or Dunnett's test, were conducted to determine specific group differences if the ANOVA results were significant. A significance level of *p*  < 0.05 was considered statistically significant for all tests performed.

## 3. Results

### 3.1. Design, Synthesis, and Characterization of SV-SeNPs

**Figure 1:**
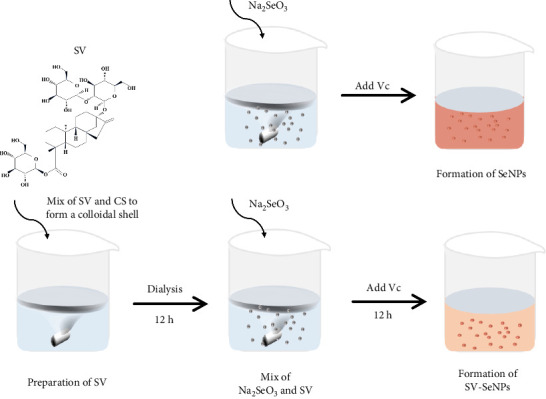
Synthetic scheme for the preparation of SeNPs and SV-SeNPs. CS, chitosan; SeNPs, selenium nanoparticles; SV, stevioside; Vc, L-ascorbic acid.

**Figure 2:**
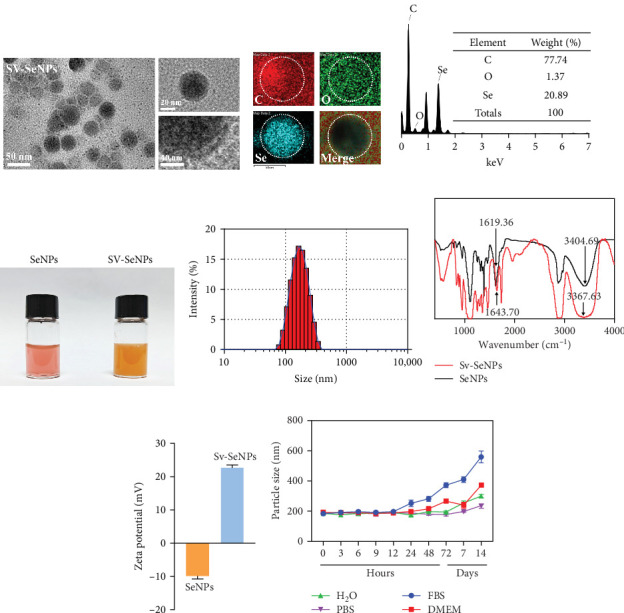
Synthesis and characterization of SV-SeNPs. (A) TEM images of SV-SeNPs. (B) EDS element mapping images of SV-SeNPs. (C) STEM-EDS elemental analysis of the MRO-SeNPs. (D) The appearance of SeNPs (left) and SV-SeNPs (right). (E) The size distribution profiles of SV-SeNPs. (F) FT-IR spectra of SeNPs and MRO-SeNPs. (G) The zeta potential of SeNPs and SV-SeNPs (*n* = 3). (H) Stability of SV-SeNPs in different media within 14 days. EDS, energy dispersive X-ray spectroscopy; SeNPs, selenium nanoparticles; SV, stevioside; TEM, transmission electron microscope.

Herein, we employ CS as a linking agent, PEG-300, and Tween-80 as macroinitiator, dispersants, and cosolvents to establish a stable Se nanosystem modified with SV under a simple redox system involving sodium selenite (Na_2_SeO_3_) and ascorbic acid (Figure 1). The field emission TEM analysis suggested that SV-SeNPs exhibited nearly spherical shapes, with sizes ranging from 40 to 80 nm (Figure 2A). Furthermore, surface elemental composition analysis of SV-SeNPs by EDS revealed the distribution of Se, C, and O elements in SV-SeNPs (Figure 2B), with the quantification showed a signal of Se atom (20.89%) from SeNPs, and C (77.74%), O (1.37%) that originated from SV (Figure 2C), confirming the successful modification of SV onto the SV-SeNPs. Visually, the appearance of SeNPs shifts from red to bright orange after SV modification (Figure 2D), and the hydrodynamic particle size distribution was 187 ± 7 nm for SV-modified SeNPs (Figure 2E). The discrepancy observed in particle size versus TEM measurements may be attributed to the pronounced hydration layer on SV-SeNPs. As displayed in Figure 2F, FT-IR spectroscopy was used to analyze the chemical bonding between the SeNPs and SV. The peaks observed at 1643.7 and 3367.63 cm^−1^ from SV-SeNPs that were shifted from 1619.36 and 3404.69 cm^−1^ for SeNPs alone and signified the formation of Se-O and Se-C bonds, respectively. This indicates a precise modification of SV on SV-SeNPs. In addition, zeta potentials of SeNPs and SV-SeNPs were measured at –9.9 ± 0.8 and 22.1 ± 0.7 mV, respectively, which implied a stronger stability of SV-SeNPs (Figure 2G). In order to assess the stability of SV-SeNPs in various media, we monitored their changes of particle size over a period of 14 days in PBS, H_2_O, DMEM, and FBS. The results showed that the size of SV-SeNPs remained stable within 72 h in H_2_O and PBS, within 48 h in DMEM and within 12 h in FBS (Figure 2H), suggesting the adequate stability under different storage and culture conditions. Taken together, these results confirmed the successful decorated of the SV on SeNPs.

### 3.2. HFP-Induced Steatosis in HepG2 Cells

**Figure 3:**
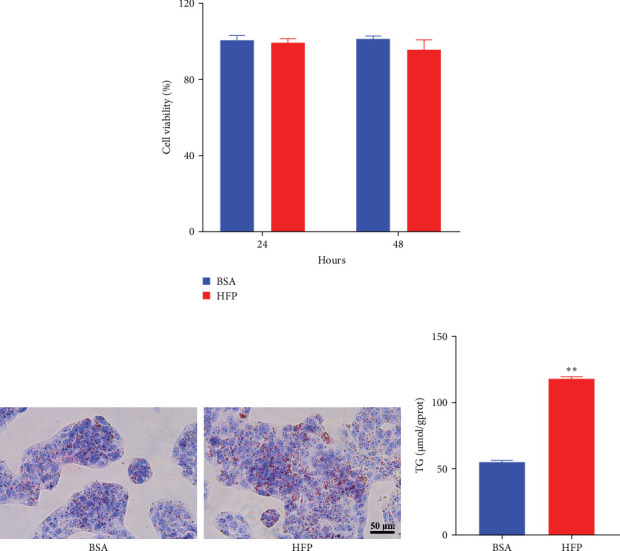
Induction of steatosis by HFP in HepG2 cells. (A) CCK8 assay of cell viability of HepG2 cells treated with different concentrations of HFP for 24 and 48 h. (B) The changes of lipid droplets in HepG2 cells after HFP-treatment for 48 h were tested by oil red O staining. (C) Determination of TG content in HepG2 cells incubated with HFP for 48 h. The data represent the mean ± SD of three independent experiments. *⁣*^*∗∗*^*p* < 0.01 indicates significant differences compared to the BSA-treated cells. HFP, high fructose-palmitate.

According to a previous study [21], the effect of HFP (100 mM fructose and 100 μM palmitate) on HepG2 cells was assessed after incubation for 48 h, and the cell viability was tested by CCK8 assay. There is no significant cell death in the HFP group (Figure 3A). Next, the results of oil red O revealed a notable increase in lipid droplet formation in HepG2 cells after HFP exposure for 48 h, in comparison to BSA group (Figure 3B). To validate the findings from the oil red O staining, we further measured the TG content in steatosis hepatocytes. The exposure to HFP resulted in a significant increase in TG accumulation in HepG2 cells when compared to the BSA group (Figure 3C).

### 3.3. SV-SeNPs Suppressed Lipid Accumulation in HFP-Treated HepG2 Cells

**Figure 4:**
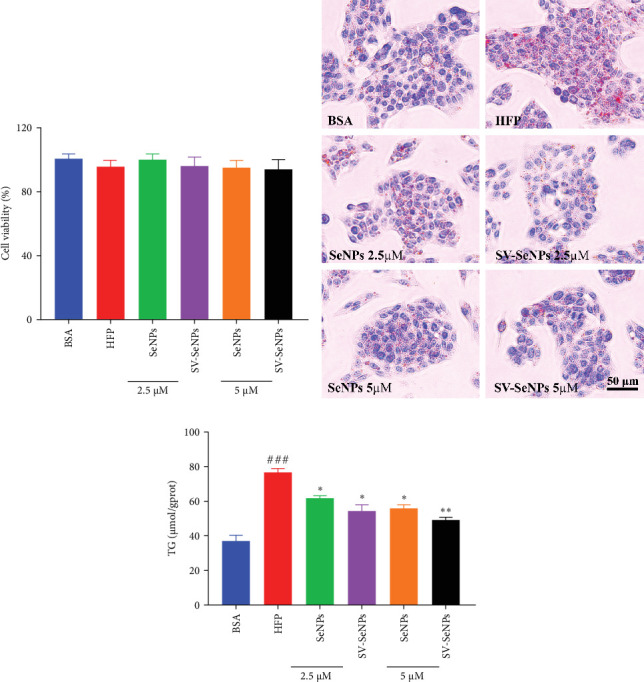
SV-SeNPs suppressed lipid accumulation in HFP-treated HepG2 cells. (A) CCK8 assay of cell viability of HFP-induced-HepG2 cells treated with different concentrations of SeNPs and SV-SeNPs for 48 h. (B) The changes of lipid droplets in HFP-induced-HepG2 cells after SeNPs and SV-SeNPs treatment for 48 h were tested by oil red O staining. (C) Determination of TG content in HFP-induced-HepG2 cells incubated with SeNPs and SV-SeNPs for 48 h. The data represent the mean ± SD of three independent experiments. ^###^*p* < 0.001 indicates significant differences compared to the BSA-treated group. *⁣*^*∗∗*^*p* < 0.01 and *⁣*^*∗*^*p* < 0.05 indicate significant differences compared to the HFP-treated group. HFP, high fructose-palmitate; SeNPs, selenium nanoparticles; SV, stevioside.

To investigate the effects of SV-SeNPs on HFP-induced lipogenesis and TG accumulation, we initially examined whether SV-SeNPs affected the cell viability of HepG2 cells. The results of CCK8 assay indicated no significant cell death following treatment with SV-SeNPs (2.5 and 5 μM) compared to BSA group (Figure 4A). Subsequent assessment of intracellular lipid accumulation was performed through oil red O staining. Compared with the BSA group, HFP exposure led to a marked increase in lipid droplet accumulation in HepG2 cells. Notably, SV-SeNPs reduced the accumulation of intracellular lipid droplets in a dose-dependent manner after incubation for 48 h compared to the HFP group (Figure 4B). Furthermore, the exposure to HFP resulted in a significant rise in TG accumulation compared to the BSA group, while SV-SeNPs reduced intracellular TG content compared to the HFP group (Figure 4C).

### 3.4. SV-SeNPs Relieve Cell Steatosis by Modulation of Lipid Metabolic Gene Expression in HFP-Treated HepG2 Cells

**Figure 5:**
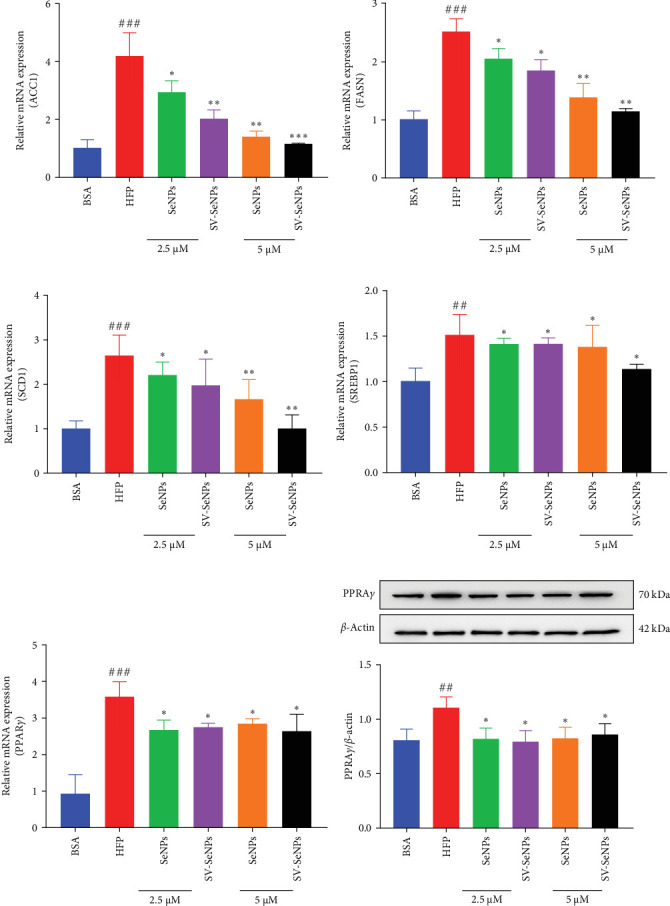
SV-SeNPs relieve cell steatosis by modulation of lipid metabolic gene expression in HFP-treated HepG2 cells. HFP-induced-HepG2 cells were incubated with SeNPs and SV-SeNPs for 48 h and followed by q-PCR analysis of the mRNA expression of (A) ACC1, (B) FASN, (C) SCD1, (D) SREBP1, (E) PPAR*γ* and western blot analysis of the protein of (F) PPAR*γ*. The data represent the mean ± SD of three independent experiments. ^###^*p* < 0.001 and ^##^*p* < 0.01 indicate significant differences compared to the BSA-treated group. *⁣*^*∗∗∗*^*p* < 0.001, *⁣*^*∗∗*^*p* < 0.01, and *⁣*^*∗*^*p* < 0.05 indicate significant differences compared to the HFP-treated group.

De novo lipogenesis plays a critical role in the regulation of hepatic lipid accumulation. To investigate the potential of SV-SeNPs to modulate the expression of endogenous de novo lipogenesis-related genes, we analyzed the effect of SV-SeNPs treatment on the mRNA expression of FASN, ACC1, and SCD1 in HepG2 cells. SV-SeNPs dramatically decreased the gene expression of ACC-1, FASN, and SCD-1 compared with HFP group (Figure 5A–C). SREBP1 serves as a key regulator of genes involved in liver lipid metabolism, influencing the expression of FASN, ACC1, and SCD1 mRNA in hepatocytes. Regarding transcription factors associated with lipid synthesis, SV-SeNPs significantly increased the gene mRNA expression of SREBP1 (Figure 5D). PPAR-*γ* mRNA levels exhibit a positive correlation with the expression of SREBP-1 [22]. The finding revealed that SV-SeNPs not only significantly decreased the mRNA level of PPAR-*γ* but also the protein level of PPAR-*γ* in HFP-treated HepG2 cells (Figure 5E,F).

### 3.5. Effects of SV-SeNPs on PI3K-AKT-Nrf2-SOD/Glutathione Peroxidase (GSHPx) Signaling Pathway and PPAR*α* in HFP-Treated HepG2 Cells

**Figure 6:**
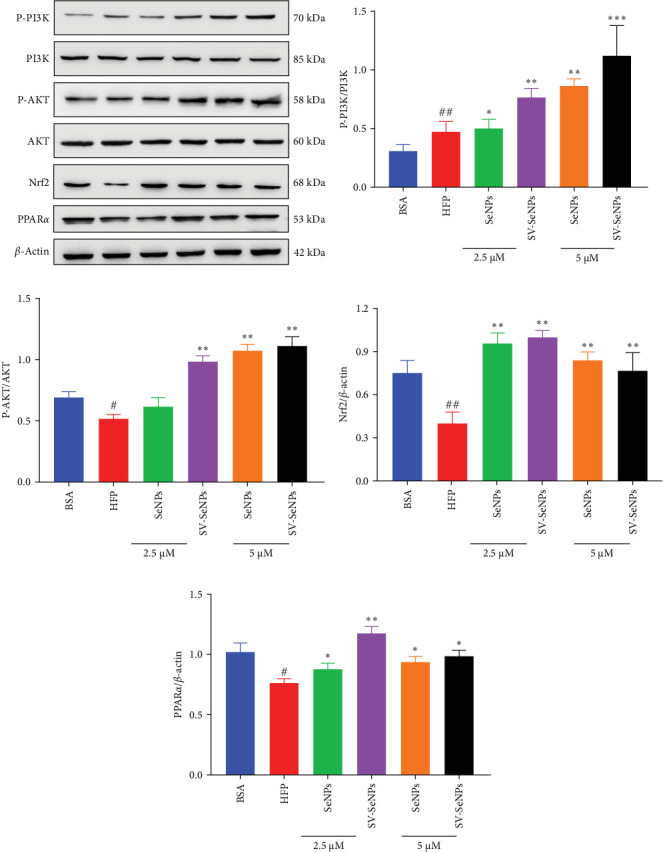
Effects of SV-SeNPs on PI3K/AKT/Nrf2 signaling pathway and PPAR*α* in HFP-treated HepG2 cells. (A) HFP-induced-HepG2 cells were incubated with SeNPs and SV-SeNPs for 48 h and followed by western blot analysis of expression of P-PI3K, P-AKT, Nrf2, and PPAR*α*. (B–E) Quantification results of the expression of P-PI3K, P-AKT, Nrf2, and PPAR*α*, respectively. ^##^*p* < 0.01 and ^#^*p* < 0.05 indicate significant differences compared to the BSA-treated group. *⁣*^*∗∗∗*^*p* < 0.001, *⁣*^*∗∗*^*p* < 0.01, and *⁣*^*∗*^*p* < 0.05 indicate significant differences compared to the HFP-treated group. HFP, high fructose-palmitate; SeNPs, selenium nanoparticles; SV, stevioside.

**Figure 7:**
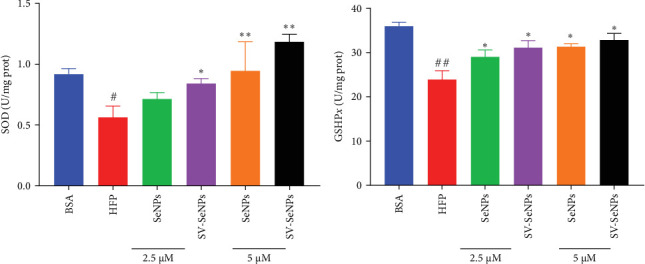
SV-SeNPs elevated the level of SOD and GSHpx in HFP-treated HepG2 cells. HFP-induced-HepG2 cells were treated with SeNPs and SV-SeNPs for 48 h, and then the intracellular SOD (A) and GSH-px (B) levels were measured according to the kit instructions. The data represent the mean ± SD of three independent experiments. ^##^*p* < 0.01 and ^#^*p* < 0.05 indicate significant differences compared to the BSA-treated group. *⁣*^*∗∗*^*p* < 0.01 and *⁣*^*∗*^*p* < 0.05 indicate significant differences compared to the HFP-treated group. HFP, high fructose-palmitate; SeNPs, selenium nanoparticles; SV, stevioside.

The activation of the PI3K/AKT/Nrf2 signaling pathway and PPAR*α* has been reported to be responsible for improvement in MAFLD [23]. Our results showed that HFP treatment decreased the expression of PI3K and AKT phosphorylation (Figure 6A,B) and suppressed the expression of Nrf2 and PPAR*α* (Figure 6C,D). Nevertheless, these effects were enhanced by SV-SeNPs in different dosages. SOD and GSHPx are two different free radical scavengers. Lower levels of SOD and GSHPx mean the body is less able to remove harmful free radicals. As depicted in Figure 7A,B, SOD and GSHpx contents were significantly lower in the HFP group compared to the BSA group. Conversely, SOD and GSHpx levels were notably elevated in SV-SeNPs groups, indicating that SV-SeNPs could improve the antioxidant capacity of the cells.

## 4. Discussion

**Figure 8:**
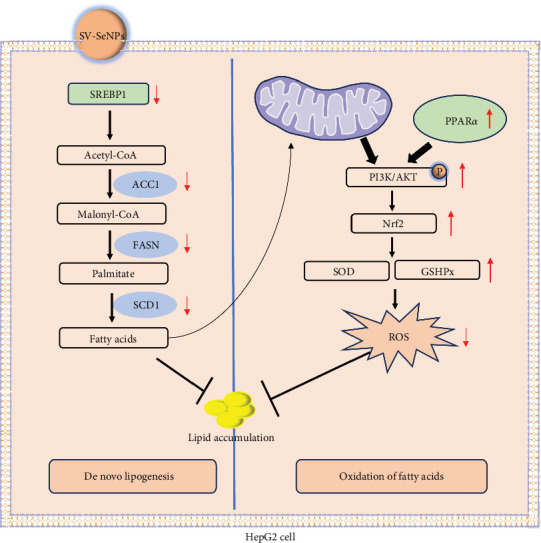
Schematic representation of the possible mechanism of SV-SeNPs mediated suppression of lipid accumulation in the HFP-induced HepG2 cells. (1) De novo lipogenesis: SV-SeNPs downregulates the expression of FASN, ACC1, and SCD1 by inhibiting SREBP1-mediated transcriptional activation, thereby reducing endogenous and fatty acid-induced lipogenesis and lipid drop accumulation in hepatocytes. (2) Fatty acid oxidation: SV-SeNPs improve fatty acid overloading induced ROS by activating the PI3K-AKT-Nrf2-SOD/GSHpx signaling pathway. HFP, high fructose-palmitate; SeNPs, selenium nanoparticles; SV, stevioside.

In this study, we successfully developed a stable, homogeneous formulation of SV-SeNPs with a consistent diameter of 187 ± 7 nm. Our research represents the first endeavor to highlight the potential role of SV in facilitating the stable dispersion of SeNPs in water. The HepG2 cells were further employed to determine the effects of SV-SeNPs on abnormal lipid accumulation and PI3K/AKT/Nrf2 oxidative stress signaling pathway, with schematic mechanism of SV-SeNPs enhancing lipid accumulation shown in Figure 8. The results showed that SV-SeNPs reduced lipid accumulation and oxidative stress. These data support the idea that SV-SeNPs may represent a promising nutrient with antilipid accumulation and antioxidant stress capabilities.

In the field of biomedicine, nanotechnology has been widely applied. Numerous studies have found that colloidal SeNPs have emerged as a special Se species with preventive and therapeutic properties. Functionalization remains a key step in the application of SeNPs in gene or drug delivery [24]. In this study, optimized synthetic SeNPs with the concentration of 2 mM could be stable under different storage and culture conditions. Size distribution is an important criterion for measuring effective NPs. Here, we found that the size distribution of SV-SeNPs is detected in the 70–100 nm range, drifting to the left of SeNPs alone. The zeta potential value of SV-SeNPs is ~22 mV, indicating that the NPs tend to repel each other and present better stability. It is worth noting that SV-SeNPs have a higher absolute zeta potential than SeNPs, indicating that SV-SeNPs have higher stability and dispersity than SeNPs alone, with no tendency to aggregate. In short, SV-SeNPs can be considered as a characteristic NP necessary for preventive and therapeutic applications.

The liver obtains lipids through the uptake of circulating fatty acids and de novo lipogenesis [25]. SREBPs are transcription factors that regulate the expression of genes involved in lipid synthesis [26]. The transcription factor SREBP1 regulates the transcription rate of genes associated with the fatty acid synthesis pathway. SREBP1 mediates the regulation of genes related to liver carbohydrate and lipid metabolism. Studies have reported that overexpression of SREBP-1c activates the lipogenic pathway and induces steatosis in hepatocytes or mice. In contrast, mice lacking SREBP-1c was unable to express lipogenic enzymes or perform TG synthesis [27]. ACC1, FASN, and SCD1 are key enzymes of de novo lipogenesis [28]. It has been reported that SREBP-1c can control the mRNA expression of FASN, ACC1 and SCD1 in hepatocytes [27, 29, 30]. In addition, expression of SREBP-1c in liver is significantly higher in MAFLD, with nearly fivefold greater than the controls [31]. In this study, we found that SV-SeNPs significantly reduced SREBP-1 mRNA levels and inhibited transcription of FASN, ACC1, and SCD1, thereby inhibiting de novo lipogenesis.

Fatty acid oxidation, which occurs primarily in mitochondria, is regulated by PPAR*α* and decreases fat levels in liver by utilizing lipids as an energy source. Fatty acids are metabolized in mitochondria mainly via peroxisome *β*-oxidation. Once fatty acid is overloaded, this process produces large amounts of reactive oxygen species (ROS) that may promote inflammation and disease progression [25]. ROS is considered to be an important factor in the progression of NAFLD [32]. Excessive accumulation of ROS could lead to mitochondrial dysfunction, liver cell injury, and lipid accumulation [33]. As the first line of defense against oxidative stress, SOD converts superoxide anion radicals into hydrogen peroxide (H_2_O_2_) and oxygen. GSHPx removes excess H_2_O_2_ by consuming GSH [34]. Therefore, the increase of SOD and GSHPx activity is an important way to alleviate liver oxidative stress. Our results show that SV-SeNPs can increase the abundance of SOD and GSHPx, inhibit the excessive production of ROS, and thus reduce the damage of hepatocytes, suggesting that SV-SeNPs can improve the HFP-induced oxidative stress of hepatocytes.

Nrf2 is a key transcription factor that regulates the expression of antioxidant proteins (SOD, GSHPx) and plays a protective role against oxidative stress in hepatocytes [35]. There were several studies showing that activation of Nrf2 through the PI3K/AKT signaling pathway significantly enhances the antioxidant capacity of hepatocytes and alleviates mitochondrial dysfunction by inhibiting NOX2 activation in HFD-fed mice [36], suggesting that PI3K/AKT/Nrf2 signaling regulates oxidative damage in hepatocytes. In order to investigate the effect of SV-SeNPs on Nrf2 signaling pathway in HFP-treated hepatocytes, western blot assay was performed and the activation of Nrf2 by SV-SeNPs was observed. At the same time, SV-SeNPs administration upregulated the phosphorylation levels of PI3K and AKT. In summary, the present results suggest that SV-SeNPs may prevent HFD-induced lipid accumulation by stimulating the PI3K-AKT-Nrf2-SOD/GSHpx signaling pathway.

Our experimental data suggest that SV-SeNPs may be a promising supplement for inhibiting lipid accumulation in hepatocytes. However, our study still has potential limitations. Both Se and SV have antioxidation and antilipid accumulation properties, and the SeNPs modified with SV are more effective than the SeNPs alone. However, the study is not bereft of potential limitations. Interrogating the intracellular mechanism through which SV-SeNPs exert their therapeutic clout necessitates further elucidation. Moreover, while in vitro cellular models provide a semblance of disease pathogenesis, they inherently lack the complexity of in vivo MAFLD models, mandating future investigations in multiple animal systems to corroborate our findings.

## 5. Conclusion

In this study, SV-SeNPs were synthesized optimally for the first time. Meanwhile, our results also showed that SV-SeNPs could reduce HFP-induced lipid droplet accumulation in HepG2 cells. The mechanism may be that SV-SeNPs downregulate the expression of genes involved in de novo lipogenesis and improve oxidative stress by activating the PI3K-AKT-Nrf2-SOD/GSHpx signaling pathway. Overall, the results of this study not only provide a strategy for functionalization to prepare nanomedicines using structurally optimized SV but also denote a feasible therapeutic option for the prevention and/or treatment of hepatic steatosis.

## Data Availability

The data supporting the findings of this study are available from the corresponding author upon reasonable request.

## Nomenclature

MAFLD: Metabolic-associated fatty liver disease
SeNPs: Selenium nanoparticles
SV: Stevioside
HFP: High fructose–palmitate
SOD: Superoxide dismutase
GSHPx: Glutathione peroxidase
NAFLD: Nonalcoholic fatty liver disease
TG: Triglyceride
Se: Selenium
SeNPs: Se nanoparticles
CS: Chitosan
Vc: L-ascorbic acid
TEM: transmission electron microscope
FASN: Fatty-acid synthase
ACC1: Acetyl-CoA carboxylase 1
SCD1: Stearoyl-CoA desaturase 1
SREBPs: Sterol regulatory-element binding proteins
ROS: Reactive oxygen species
H_2_O_2_: Hydrogen peroxide.

## Supporting Information

Supporting Information Table S1. Antibodies used in this study. Table S2. Set of primers used in this study.
