# Supplementary material for: Selenium Nanoparticles Decorated With Stevioside Potentially Attenuate Fructose Palmitate Induced Lipid Accumulation in HepG2 Cells
Source: Mediators Inflamm. 2025 Feb 13;2025:7942947. doi: 10.1155/mi/7942947 (PMC11842145; doi:10.1155/mi/7942947)
Supplement: Supporting Information — Table S1. Antibodies used in this study. Table S2. Set of primers used in this study. [file 7942947.f1.docx]

**Supporting Information**

| **Antibody Name** | **Catalog Number** | **Source** |
| --- | --- | --- |
| β-actin | BM0627 | Boster Biological Technology, China |
| AKT | # 4691 | Cell Singling Technology, USA |
| Phospho-Akt (Ser473) | # 4060 | Cell Singling Technology, USA |
| PPARα | 66826-1 | Proteintech, China |
| PPARγ | 22061-1-AP | Proteintech, China |
| Nrf2 | 16396-1-AP | Proteintech, China |
| Phospho-PI3K | AF5905 | Beyotime, China |
| PI3K | AF7742 | Beyotime, China |
| HRP-conjugated Affinipure Goat Anti-Rabbit IgG(H+L) | SA00001-2 | Proteintech, China |
| HRP-conjugated Affinipure Goat Anti-Mouse IgG(H+L) | SA00001-1 | Proteintech, China |

**Supplementary Table 1: Antibodies used in this study**

| ACC1 F: 5’- AGTGAGGATGGCAGCTCTGGA-3’  ACC1 R: 5’- TGAGATGTGGGCAGCATGAAC-3’ |
| --- |
| SREBP1 F: 5’-GAGCCATGGATTGCACTTTC-3’  SREBP1 R: 5’-AGCATAGGGTGGGTCAAATAG-3’ |
| SCD1 F: 5’- AAGTGCCTCACCTCGAAAGG-3’  SCD1 R: 5’- TGAGATGTGGGCAGCATGAAC-3’ |
| FASN F: 5’- GCAAGCTGAAGGACCTGTCT -3’  FASN R: 5’- AATCTGGGTTGATGCCTCCG -3’ |
| PPARγ F: 5’- CCAGAAGCCTGCATTTCTGC -3’  PPARγ R: 5’- CGAGCTCCAAGCTACTGTCC -3’ |
| PPARα F: 5’- ACCCATGACACCCTCGTCTA -3’  PPARα R: 5’- CGAGCTCCAAGCTACTGTCC -3’ |
| GAPDH F: 5’- CCTGCACCACCAACTGCTTA -3’  GAPDH R: 5’- AGTGATGGCATGGACTGTGG -3’ |

**Supplementary Table 2:** **Set of primers used in this study**
